# Early primed KLRG1^-^ CMV-specific T cells determine the size of the inflationary T cell pool

**DOI:** 10.1371/journal.ppat.1007785

**Published:** 2019-05-13

**Authors:** Nicolas S. Baumann, Suzanne P. M. Welten, Nicole Torti, Katharina Pallmer, Mariana Borsa, Isabel Barnstorf, Jennifer D. Oduro, Luka Cicin-Sain, Annette Oxenius

**Affiliations:** 1 Institute of Microbiology, ETH Zürich, Vladimir-Prelog-Weg 4, Zürich, Switzerland; 2 Department of Vaccinology and Applied Microbiology, Helmholtz Centre for Infection Research, Braunschweig, Germany; University of Wisconsin-Madison, UNITED STATES

## Abstract

Memory T cell inflation is a process in which a subset of cytomegalovirus (CMV) specific CD8 T cells continuously expands mainly during latent infection and establishes a large and stable population of effector memory cells in peripheral tissues. Here we set out to identify *in vivo* parameters that promote and limit CD8 T cell inflation in the context of MCMV infection. We found that the inflationary T cell pool comprised mainly high avidity CD8 T cells, outcompeting lower avidity CD8 T cells. Furthermore, the size of the inflationary T cell pool was not restricted by the availability of specific tissue niches, but it was directly related to the number of virus-specific CD8 T cells that were activated during priming. In particular, the amount of early-primed KLRG1^-^ cells and the number of inflationary cells with a central memory phenotype were a critical determinant for the overall magnitude of the inflationary T cell pool. Inflationary memory CD8 T cells provided protection from a Vaccinia virus challenge and this protection directly correlated with the size of the inflationary memory T cell pool in peripheral tissues. These results highlight the remarkable protective potential of inflationary CD8 T cells that can be harnessed for CMV-based T cell vaccine approaches.

## Author summary

Cytomegalovirus induces a lifelong infection in the majority of the world's population, due to the ability of the virus to establish latency. Upon CMV infection, large numbers of effector memory T cells are induced in peripheral tissues, a process that is termed memory inflation. As inflationary T cells are highly functional, CMV-based vaccines have gained substantial interest for vaccination purposes. Here we examine factors that promote and limit memory T cell inflation. We found that there were no constraints on the availability of specific niches for inflationary T cells in tissues and that high avidity T cells predominately contribute to the inflationary T cell population in the beginning of infection. Moreover, the number of early primed KLRG1^-^ CMV-specific T cells in the acute phase of infection set the limit for memory T cell inflation. Furthermore, we show that inflationary T cells provided protection from a pathogenic challenge in peripheral tissues such as the ovaries. Thus, inflationary T cells comprise a population of T cells that can protect peripheral tissues from pathogenic infections and their efficacy can be regulated by balancing the number of KLRG1^-^ CMV-specific cells during priming.

## Introduction

A hallmark of immunological memory is the ability of the adaptive immune system to generate long-lived antigen-specific memory T or B cells. Upon pathogen clearance, most virus-specific T cells undergo apoptosis and few of them form a stable pool of memory T cells, which is maintained lifelong in case of CD8 T cells. Pre-existing memory T cells are beneficial for protection against reinfection with the same pathogens, since they are numerically increased compared to naive antigen-specific T cells, have widened anatomical distribution and respond quickly by vigorous proliferation and acquisition of effector functions, conferring rapid clearance of the infectious agent. Long after resolution of acute viral infection, memory T cells reside primarily in lymphoid tissues as central memory cells [1] until they re-encounter their cognate antigen, with the exception of tissue-resident memory cells that have acquired long-term tissue residence and are largely disconnected from recirculation [2]. In chronic active virus infections, with abundant presence of viral antigens, formation of antigen-experienced memory cells that are long-term maintained in absence of antigen is impaired and virus-specific CD8 T cells exhibit a gradual loss of effector functions, known as T cell exhaustion [3, 4]. However, during latent reactivating virus infections, such as in the case of herpes virus infection, viruses go into latency with limited/ absent expression of viral proteins. However, sporadic viral reactivation events can occur in response to various external stimuli [5, 6], leading to reactivation of the lytic program and hence to expression of viral proteins whose peptides will be presented to CD8 T cells. This leads to sporadic reactivation and stimulation of memory CD8 T cells with specificity for those antigens, resulting in a pool of functional effector-like and not exhausted CD8 T cells [7–10].

One representative of this family of herpesviruses is cytomegalovirus (CMV), a ubiquitous β-herpesvirus. The CD8 T cell response induced by CMV is atypical, as a subset of CMV-specific CD8 T cells shows little decline after initial expansion and continues to increase in size to establish a large pool of effector memory T cells that preferentially localize to peripheral tissues [10, 11]. This phenomenon of gradual accumulation of some CMV-specific memory CD8 T cells has been termed "memory inflation" [8, 10–13]. Like in conventional CD8 T cell responses, the majority of inflationary CD8 T cells are primed during acute murine cytomegalovirus (MCMV) infection by cross-presenting dendritic cells (DCs), however, they are subsequently reactivated by antigen presentation on latently infected non-hematopoietic cells [14–17], promoting their accumulation during viral latency. The size of the inflationary T cell pool becomes remarkably large and can reach up to 50% of the whole CD8 T cell pool in an infected individual [18, 19]. The mechanisms underlying inflation of certain CMV-specific CD8 T cells are still poorly understood. Previous studies have implicated the importance of the location of the epitope within the CMV genome and within the protein context, and the dependence on the constitutive proteasome for antigen processing [20, 21].

With the emerging interest in the design of T cell-based vaccines, CMV-based vectors have gained a lot of interest due to their ability to induce these large pools of peripheral effector memory CD8 T cells. Accordingly, CMV-based vectors have been very successfully used for the induction of potent CD8 T cell responses that mediate protection against viral challenge infections and even tumors [22–27]. Due to this capacity, it is important to better understand the driving and limiting factors that shape the inflationary CD8 T cell pool in order to optimize memory CD8 T cell responses in the context of CMV-based vaccines.

In this study, we addressed the question of how memory inflation is regulated in its composition and size after resolution of lytic MCMV infection. Our data shows that the inflationary CD8 T cell pool consists of high-avidity CD8 T cells. We further demonstrate that the stable size of the inflationary T cell pool in peripheral tissues is not limited by local niches but rather that early primed MCMV-specific cells established during acute infection directly correlate with the size of the inflationary T cell pool during latency. By modulating either the size of early primed cells during CMV infection, or the amount of T_CM_ cells during viral latency, we were able to accordingly modulate the size of the inflationary memory CD8 T cell pool. Moreover, we show that the inflationary T cell pool provides protection from a peripheral virus re-challenge and the ability to control peripheral viral replication was correlated with the size of the inflationary T cell pool.

## Results

### High avidity T cells mainly contribute to memory inflation

**Fig 1:**
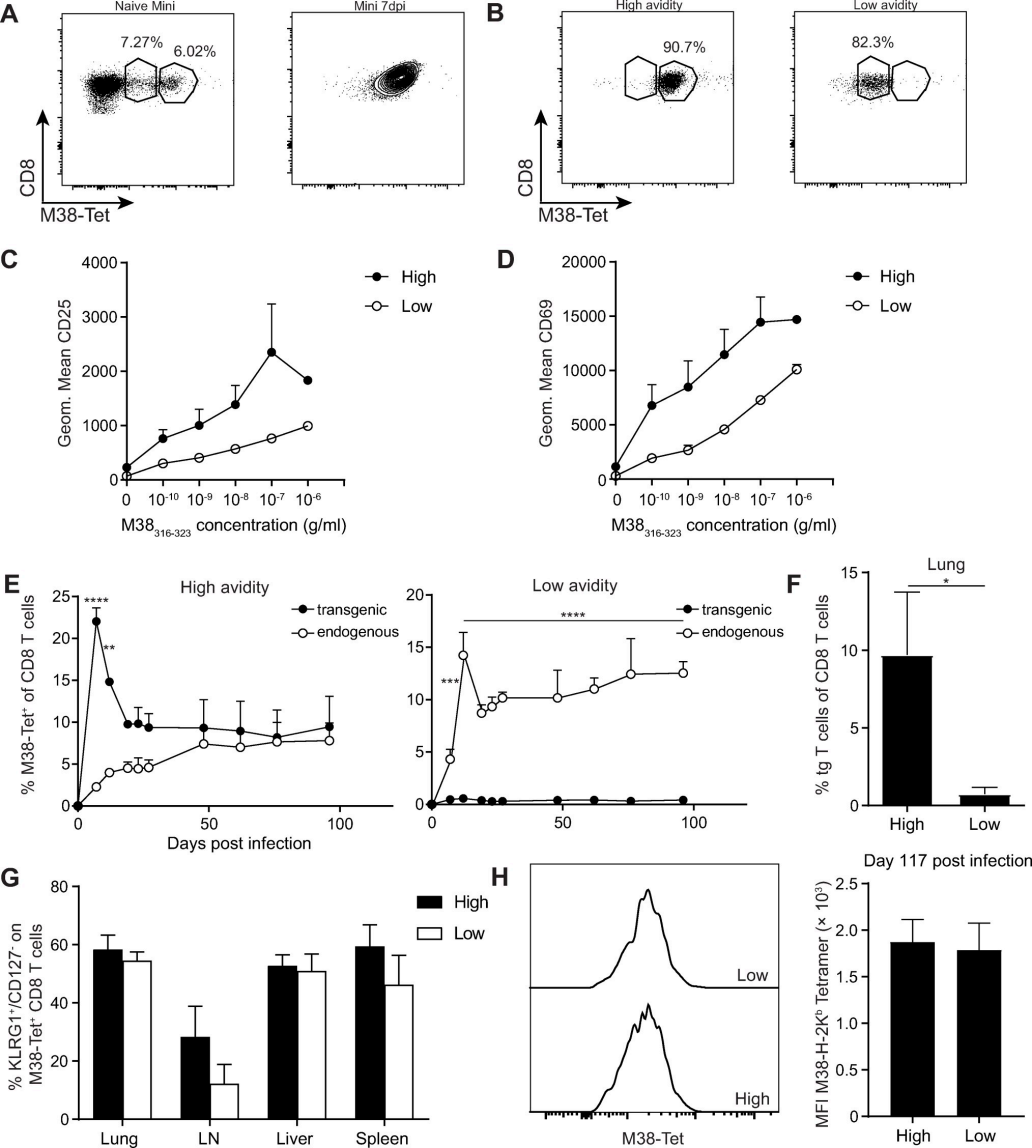
High avidity Mini cells supply the inflationary T cell pool. 10^5^ sorted low and high avidity CD45.1^+^ Mini CD8 T cells were adoptively transferred into naïve C57BL/6J mice prior to an i. v. infection with 5 × 10^6^ PFU MCMVΔm157. (A) Representative contour plot showing naïve Mini CD8 T cells (left plot) and Mini cells (gated on CD8^+^ CD45.1^+^) from a day 7 infected host after adoptive transfer (right plot). The percentages within CD8 T cells are indicated. (B) Sorted high (left plot) and low avidity (right plot) Mini CD8 T cells according to M38-tetramer staining intensity are shown. (C+D) Sorted naïve high and low avidity Mini cells were exposed to various concentrations of M38_316-323_ peptide. The geometric means of CD25 (C) and CD69 (D) are shown as mean + SEM. (E) Percentages of M38-specific CD8 T cells were measured in the blood of mice transferred with high (left graph) or low avidity Mini cells (right graph). (F) Percentages of low and high avidity Mini cells in the lungs at day 117 post infection are shown as mean + SEM of n = 3–4 mice, representative of two independent experiments. (G) Percentages of KLRG1^+^ CD127^-^ expression on Mini cells in the lungs and spleen at day 117 post infection are shown as mean + SEM of n = 3–4 mice representative of two independent experiments. (H) Staggered overlay of histograms of mean fluorescence intensities of M38-tetramer (left). Mean fluorescence intensities on low and high avidity Mini cells in the lungs at day 117 post infection are shown as mean + SEM of n = 3–4 mice representative of two independent experiments. (E-H) *p<0.05; **p<0.01; ***p<0.001; ****p<0.0001. Statistical analyses were performed using two-way ANOVA followed by Sidak's multiple comparisons test (E) or the unpaired two-tailed Student's *t* test (F-H).

T cell receptors (TCR) exhibit different avidity to a certain MHCI-antigen complex, and usually T cells recognising their cognate antigen with high avidity will be activated and contribute strongly to an effective cytotoxic CD8 T cell response. As low avidity CD8 T cells were reported to contribute to memory inflation during HCMV infection [28], we addressed the question whether TCR avidity plays a role for memory inflation in MCMV infection. We made use of the TCR beta chain transgenic (tg) Mini mouse [15], in which all T cells express the Vβ10Jβ2.1 chain of an M38_316-323_-specific TCR in combination with endogenous alpha chains, and approximately 10% of these naive CD8 T cells bind the MCMV-specific H-2K^b^/M38_316-323_ tetramer, albeit with a broad range of staining intensity, indicative of variable avidities (Fig 1A). Upon adoptive transfer and MCMV infection (MCMVΔm157 was used, hereafter referred to as MCMV), Mini TCR tg CD8 T cells expanded vigorously, resulting in a large population of M38-tetramer^+^ cells with apparent high avidity, based on tetramer staining intensity (Fig 1A). To address whether high or low avidity Mini CD8 T cells contributed to the acute and / or inflationary response, we sorted naïve Mini TCR beta transgenic CD8 T cells into a population with low or high tetramer staining, indicative of lower and higher avidity for the M38_316-323_ peptide H-2K^b^ complex (Fig 1B). To corroborate that low tetramer binding Mini CD8 T cells can in fact react towards their cognate antigen, we exposed high and low tetramer binding Mini CD8 T cells to a range of different M38_316-323_ peptide concentrations. Both populations of Mini cells upregulated the early T cell activation markers CD25 (Fig 1C) and CD69 (Fig 1D), indicative of TCR stimulation, yet the high tetramer binding Mini cells had increased levels of these activation markers compared to their low tetramer binding counterparts, indicating that the low tetramer binding population has a lower functional avidity as compared to the high tetramer binding population. The low and high avidity subpopulations were adoptively transferred into separate naïve recipients, followed by MCMV infection. We analysed the frequencies of transgenic and endogenous M38-specific CD8 T cell responses longitudinally in the blood (Fig 1E). Mice receiving high avidity Mini CD8 T cells showed a strong contribution of the transgenic Mini CD8 T cells to the overall M38-specific T cell response with a long term contribution of 50% (Fig 1E, left graph). In contrast, low avidity Mini T cells contributed only marginally to the overall M38-specific CD8 T cell response in the blood (Fig 1E, right graph) as well as in peripheral organs such as the lungs (Fig 1F). Regardless of their very low frequencies, these low avidity Mini cells exhibited an activated phenotype (KLRG1^+^/CD127^-^) alike their high avidity counterparts (Fig 1G). Furthermore, despite the obviously reduced numbers of low avidity M38-specific CD8 T cells, we found no significant differences in the mean fluorescence intensities of the M38-tetramer staining (Fig 1H). This indicates that the activated CD8 T cells from the low avidity population might actually be the ones with relative higher avidities, whereas the "really" low avidity cells were outcompeted by endogenous higher avidity M38-specific CD8 T cells. Similar observations were made using OT-III cells [29]. These cells, similar as OT-I T cells, are specific for the SIINFEKL epitope of Ovalbumin; however, OT-III cells express a low avidity TCR as compared to OT-I T cells. Both OT-I (CD45.1^+^) and OT-III (CD90.1^+^) cells were exposed to an MCMV virus expressing the SIINFEKL epitope under the immediate early 2 (*ie*2) promoter [21]. Of note, this virus expresses the viral protein m157 that binds to the NK cell receptor Ly49H and thereby activates Ly49H^+^ NK cells early in MCMV infection [30, 31]. No significant contribution of the OT-III cells to the acute or inflationary response was observed in blood (S1A Fig, S1B Fig) or in organs (S1C Fig). Yet, the few recovered OT-III cells exhibited a similar phenotype as their high affinity OT-I counterparts (S1D Fig). These data show in addition that in the presence or absence of the MCMV m157 protein mainly high avidity T cells are recruited into the MCMV-specific response. As OT-I and OT-III T cells are monoclonal T cell populations, the difference in SIINFEKL-H2-K^b^ tetramer binding, indicative for T cell avidity, were still observed at day 60 post infection (S1E Fig). These findings imply that high avidity CD8 T cells mainly contribute to the inflationary T cell pool—at least within the first 100 days of infection—whereas low avidity CD8 T cells do not seem to be a major contributor to the large pool of inflationary cells during MCMV latency.

### "Space" in peripheral tissues is not a limiting factor for memory inflation

**Fig 2:**
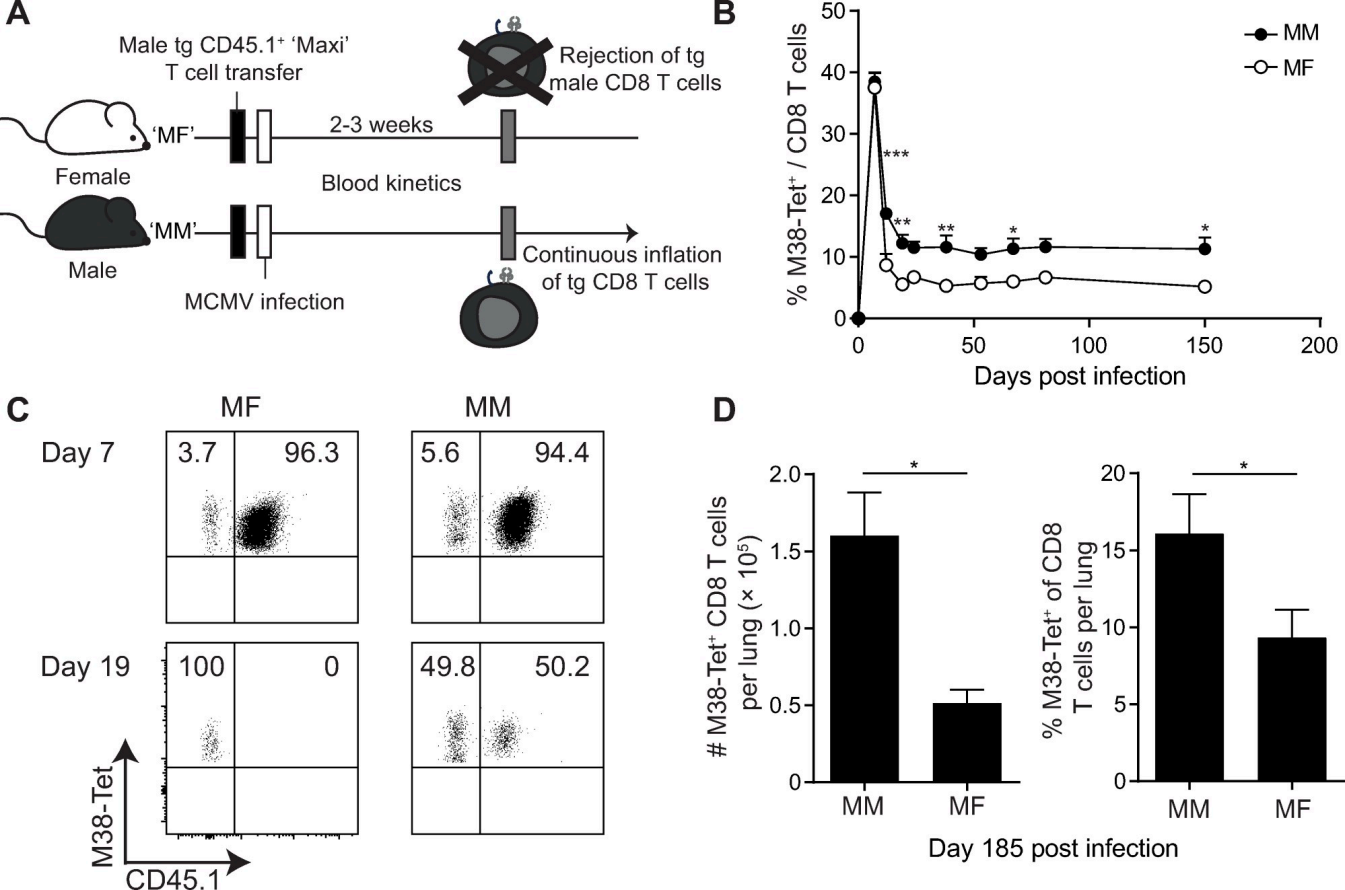
Space does not limit the size of the inflationary T cell pool. (A) Experimental setup: 10^4^ male or female CD45.1^+^ Maxi CD8 T cells were transferred into naïve female C57BL/6 mice one day prior to i. v. infection with 5 × 10^6^ PFU MCMVΔm157. (B) Percentages of M38-specific (endogenous and Maxi) CD8 T cells in mice transferred with male or female Maxi cells were measured in the blood. (C) Representative flow cytometry plot shows the percentage of endogenous (CD45.1^-^) and transgenic (CD45.1^+^, Maxi) M38-specific cells in the blood at day 7 and day 19 post MCMV infection. M38-specificity was determined by tetramer staining. (D) Total numbers and percentages of M38-specific CD8 T cells in the lungs at day 185 post infection are shown as mean + SEM of n = 5 mice representative of two independent experiments. (B and D) *p<0.05; **p<0.01; ***p<0.001. Statistical analyses were performed using two-way ANOVA followed by Sidak's multiple comparisons test (B) or the unpaired two-tailed Student's *t* test (D).

So far, we have defined a parameter that contributes to fuel memory inflation, which is expression of a high avidity TCR with specificity to an epitope that drives memory inflation. Despite this driving force, inflationary M38-specific CD8 T cells stabilize at about 10% of CD8 T cells in blood and lung tissue [8, 15, 33], raising the question of what limits memory inflation in peripheral tissues. We hypothesized that there might be limited "space", defined by survival niches for inflationary CD8 T cells in peripheral tissues, such as provision of IL-15 in lung tissue [33]. To test this hypothesis, we used an experimental system in which the CD8 T cell population with inflationary specificity is curtailed by 50% after initial clonal expansion, thereby creating "new space". If space limitations would limit memory inflation, we reasoned, this vacated space should be re-occupied to regain the level of 10% of M38-specific CD8 T cells within the CD8 T cell population in blood and lungs. For this purpose, sex mismatched adoptive transfer experiments were performed, in which naïve male transgenic CD45.1^+^ Maxi CD8 T cells [15] were adoptively transferred into female recipients, followed by MCMV infection (Fig 2A). Maxi cells are a monoclonal population of TCR transgenic T cells that all express Vα4Jα13 and Vβ10Jβ2.1. In contrast to the Mini cells, where only 10% is specific for the M38_316-323_ peptide due to the usage of endogenous TCR α-chains, all Maxi CD8 T cells are specific for the M38_316-323_ peptide of MCMV [15]. In this setting, priming of both endogenous and transferred Maxi cells occurred (Fig 2B, Fig 2C) and in the sex-matched hosts, the population of M38-specific CD8 T cells consisted to 50% of endogenous and 50% of Maxi CD8 T cells at day 19 post MCMV infection (Fig 2B, Fig 2C). In the sex-mismatched hosts, the male Maxi cells were rejected between 2 and 3 weeks of infection, thereby halving the total population of M38-specific CD8 T cells (Fig 2B, Fig 2C). If there would be a space limitation, we expected that endogenous M38-specific CD8 T cells would converge to the levels that were observed in sex-matched hosts. Yet, we did not see such a convergence (Fig 2B) neither in blood, nor lungs (Fig 2D), indicating that "space" did not limit M38-specific memory CD8 T cell inflation. We performed similar experiments (S2A Fig) using TCR beta-chain transgenic Mini cells (containing roughly 10% CD8 T cells specific for the M38_316-323_ epitope) for adoptive transfer, and observed a similar pattern: In female hosts, the rejection of male M38-specific CD8 T cells occurred two to three weeks post infection and the created "space" was not replenished by endogenous M38-specific CD8 T cells (S2B Fig, S2C Fig). To exclude that CD8 T cells with different MCMV-specificities would fill the newly available space, we quantified MCMV-specific CD8 T cells specific for the M45_985-993_ (non-inflationary), IE3_416-423_ or m139_419-426_ (inflationary) epitopes, and did not observe any increased frequencies in the lungs of sex-mismatched hosts (S2D Fig). Taken together, these data suggest that "space" does not seem to be a limiting factor for the inflationary T cell pool in blood, spleen or peripheral tissues.

### Increase of T_CM_ cells leads to larger pools of inflationary CD8 T cells

**Fig 3:**
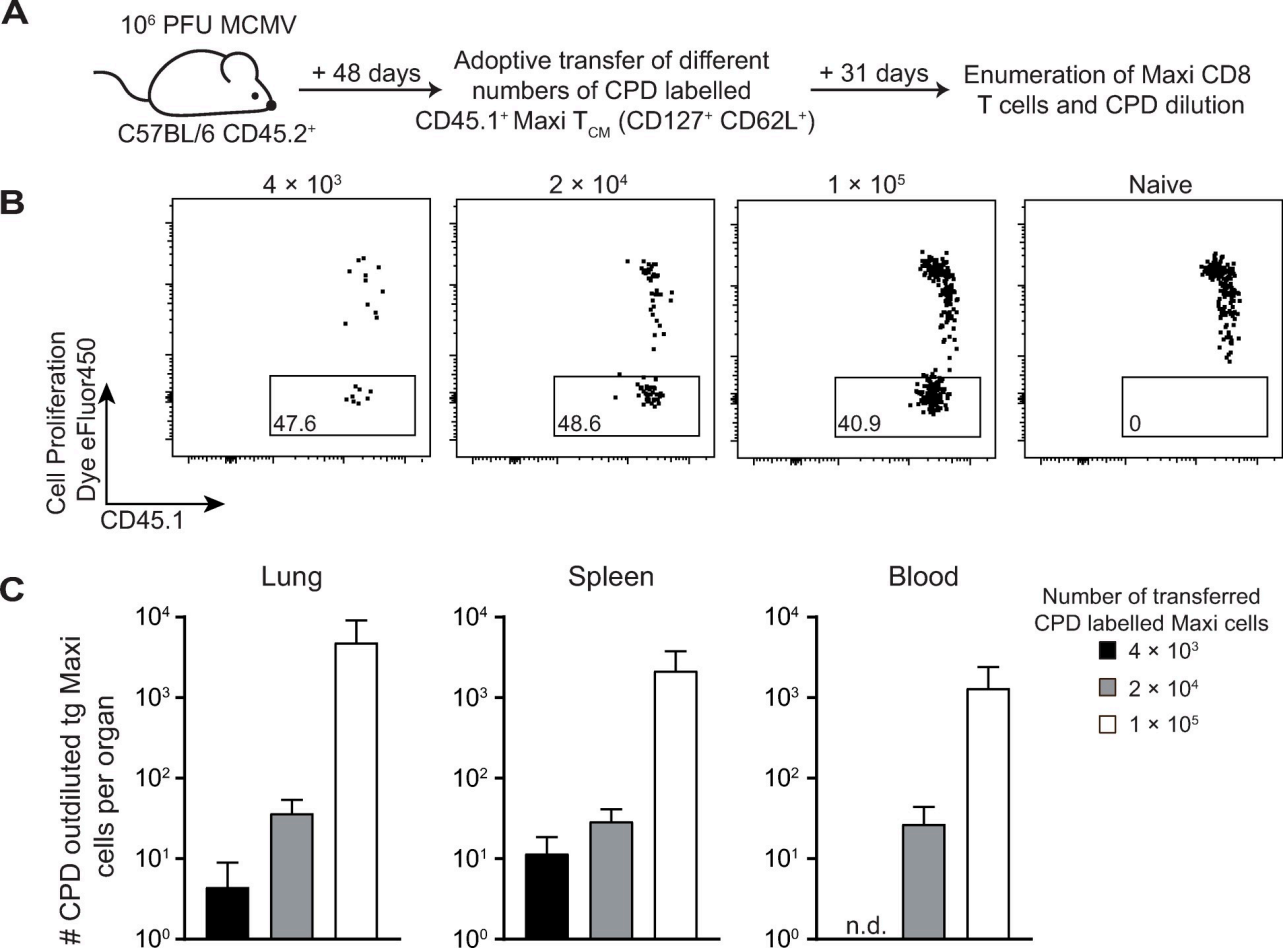
Transfer of higher amounts of Maxi T_CM_ cells leads to increased numbers of peripheral inflationary T cells. (A) Experimental setup: gradual numbers (4 × 10^3^, 2 × 10^4^ or 1 × 10^5^) of CD45.1^+^ T_CM_ (CD127^+^ /CD62L^+^) Maxi cells were labelled with cell proliferation dye-eFluor450 (CPD) and adoptively transferred into mice that were infected with MCMV for 48 days. Thirty-one days post transfer, Maxi cells were enumerated and the proliferation history was determined. (B) Representative flow cytometric dot plot shows the proliferation profile of T_CM_ Maxi cells in the spleen 31 days post transfer into MCMV infected hosts. Naïve mice received 10^5^ Maxi cells. Numbers indicate the percentage of Maxi cells that have completely out-diluted the proliferation dye. Cells are gated on CD45.1 expression. (C) Bar graphs show the number of Maxi cells that have out-diluted the proliferation dye in lungs, spleen and blood as mean + SEM, n = 2–4 mice per group, representative of two independent experiments.

During latent MCMV infection, a small fraction of the M38-specific T cell population has a central memory phenotype (T_CM_, CD62L^+^/CD127^+^/KLRG1^-^) and this population is markedly enriched in lymph nodes [15]. One hypothesis is that these T_CM_ cells are able to sense viral antigens derived from viral reactivation events in non-hematopoietic cells and provide newly activated T cells that supply the inflationary T cell pool [15]. To demonstrate that T_CM_ cells have the potential to fuel the pool of inflationary CD8 T cells, we adoptively transferred graded doses of central memory Maxi cells into host mice that were latently infected with MCMV. In addition, these transferred Maxi T_CM_ cells were labelled with a cell proliferation dye in order to track cell division (Fig 3A). The percentage of Maxi cells that had out-diluted the proliferation dye was comparable in all conditions (Fig 3B), indicative of comparable antigen encounter on latently infected cells. Furthermore, cells that had out-diluted the cell proliferation dye were not found in uninfected mice (Fig 3B). Adoptive transfer of increasing numbers of Maxi T_CM_ cells resulted in increasing numbers of Maxi cells having out-diluted the proliferation dye in the spleen, lungs and blood 31 days post transfer (Fig 3C). These data demonstrate that not only T_CM_ cells contribute to the peripheral inflationary T cell pool but also that their number correlates with the size of the inflationary T cell pool.

### The precursor frequency of MCMV-specific T cells determines the degree of memory T cell inflation

**Fig 4:**
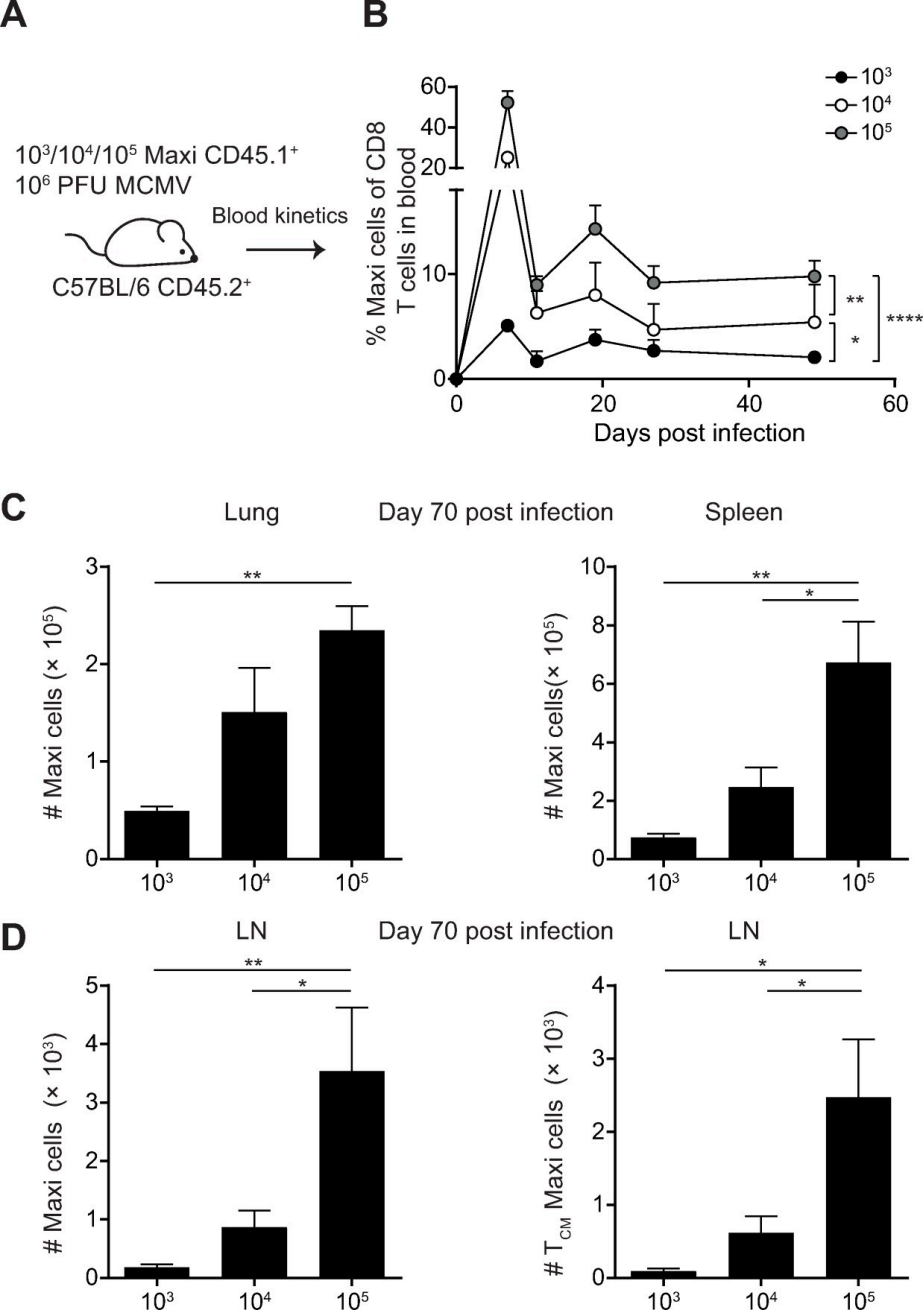
The precursor frequency of MCMV-specific CD8 T cells impacts memory T cell inflation. (A) Experimental setup: Naïve C57BL/6 mice were transferred with 10^3^, 10^4^ or 10^5^ CD45.1^+^ Maxi CD8 T cells one day prior to i. v. infection with 10^6^ PFU MCMVΔm157. (B) Percentages of Maxi cells within CD8 T cells were measured in the blood at indicated time points post infection. (C) Total numbers of Maxi cells in the lungs and spleen at day 70 post infection are shown as + SEM of n = 5 mice representative of two independent experiments (D) Total numbers of Maxi cells (left graph) and Maxi T_CM_ cells (CD127^+^/KLRG1^-^) (right graph) in the LN at day 70 post infection are shown as mean + SEM of n = 5 mice representative of two independent experiments. (B-D) *p<0.05; **p<0.01, ****p<0.0001. Statistical analyses were performed using the one-way ANOVA using Tukey's multiple comparisons test (C-D) or two-way ANOVA followed by Sidak's multiple comparisons test (B, significance is only indicated for the final time point).

As the number of transferred T_CM_ cells during latent MCMV infection correlated with emerging size of activated T cells, we addressed whether the number of MCMV-specific T cells that is primed during MCMV infection influences the extent of memory T cell inflation. We experimentally increased M38-specific T cells by adoptively transferring different numbers of naïve Maxi CD8 T cells into hosts one day prior to MCMV infection (Fig 4A). Increasing the number of adoptively transferred Maxi CD8 T cells resulted in heightened peak clonal expansion in the acute phase of MCMV infection (Fig 4B). The transfer of different numbers of Maxi cells also translated into corresponding differences in the percentage of Maxi cells during latent MCMV infection (day 70) in the blood (Fig 4B). Furthermore, the total number of Maxi cells in the LN, lungs and spleen was significantly increased when more cells were transferred (Fig 4C, Fig 4D). The amount of T_CM_ cells in the LN (day 70) was also increased when more Maxi cells were transferred (Fig 4D). The same pattern was observed in experiments where different numbers of OT-I CD8 T cells were adoptively transferred and the hosts were infected with MCMV-*ie2*-SIINFEKL (an MCMV virus that expresses the viral protein m157) (S3 Fig). Taken together, these results show that the precursor frequency of MCMV-specific T cells directly correlates with the size of the inflationary CD8 T cell pool.

### The number of early primed KLRG1^-^ MCMV-specific T cells correlates with the size of memory inflation

**Fig 5:**
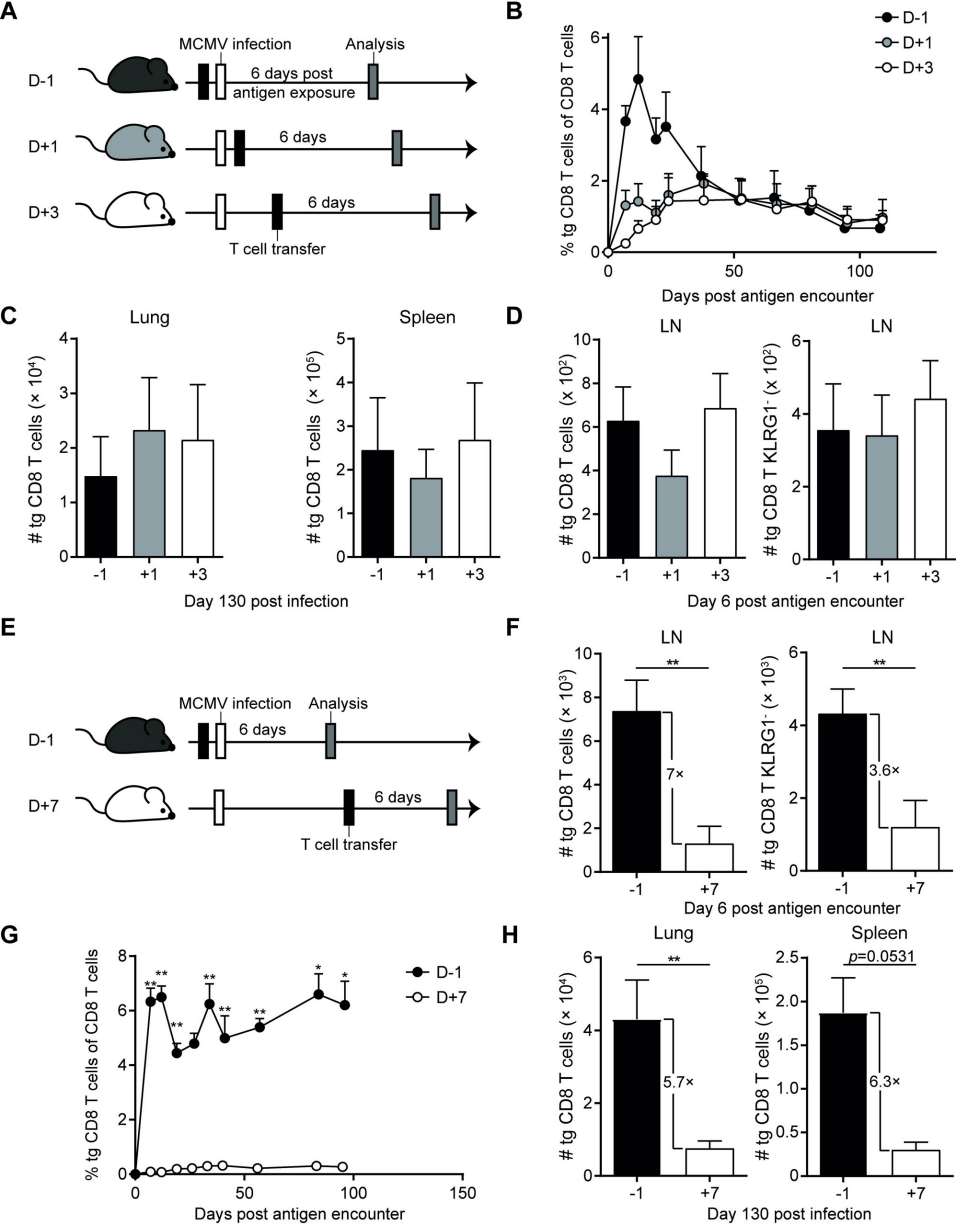
Early responding M38-specific T cells predict the size of memory inflation. (A) Experimental setup: 2 × 10^5^ CD45.1^+^ Mini CD8 T cells were adoptively transferred into C57BL/6 mice one day prior, or one or three days after an i. v. infection with 5 × 10^6^ PFU MCMVΔm157. (B) Percentages of Mini cells among CD8 T cells were determined in the blood at indicated days post transfer. (C) Total numbers and percentages of M38-specific CD8 T cells in the lungs and spleen at day 130 post infection are shown as mean + SEM of n = 5 mice representative of two independent experiments. (D) Total numbers of Mini and Mini KLRG-1^-^ cells in the LN six days post antigen exposure are shown as mean + SEM of n = 5 mice representative of three independent experiments. (E) Experimental setup: 2 × 10^5^ CD45.1^+^ Mini CD8 T cells were adoptively transferred in naïve C57BL/6 mice one day prior or seven days after i. v. infection with 5 × 10^6^ PFU MCMVΔm157. (F) Total numbers of Mini and KLRG1^-^ Mini cells in the LN six days post antigen exposure are shown as mean + SEM of n = 10 mice pooled from two independent experiments. (G) Frequencies of Mini cells within CD8 T cells were determined in the blood at indicated time points post transfer and are shown as mean + SEM of n = 5 mice, representative of three experiments. (H) Total numbers and percentages of Mini CD8 T cells in the lungs and spleen at day 130 post infection are shown as mean + SEM of n = 15–25 mice, pooled from three independent experiments. Statistical analyses were performed using the non-parametric Mann-Whitney *U* test. (F, H) *p<0.05; **p<0.01.

Our previous results indicated that both the precursor frequency prior to infection, and the number of T_CM_ cells during established latent infection, correlate with the extent of memory inflation. In acute LCMV infection, early primed CD127^+^/KLRG1^-^ cells have a higher probability to feed into the memory pool [34]. We therefore speculated that the number of KLRG1^-^ M38-specific cells that are established early during MCMV infection might relate to the size of the inflationary pool. We addressed this hypothesis by adoptively transferring M38-specific Mini CD8 T cells at different time points relative to the onset of MCMV infection, thereby creating different ratios of KLRG1^+^ and KLRG1^-^ cells, with late recruited CD8 T cells preferentially adopting KLRG1^-^ phenotypes [32] (S4 Fig). We transferred Mini CD8 T cells one day prior and one or three days after MCMV infection (Fig 5A). Despite large differences in the size of clonal expansion in the blood, Mini cells reached similar percentages by day 50 post antigen encounter (Fig 5B) and equivalent numbers of Mini cells were present in spleen and in the lungs at 130 days post infection (Fig 5C). Thus although there were large differences in the magnitude of clonal expansion, a similar level of memory inflation was achieved, indicating that it is not just the overall number of MCMV-specific T cells in the acute phase of infection that sets the limit for memory inflation. Strikingly, 6 days post antigen encounter the numbers of total Mini and KLRG1^-^ Mini cells in the LNs did not differ between the three groups (Fig 5D). These data imply that the number of primed Mini cells, established early in the lymph node, correlates with the size of memory T cell inflation.

As no differences were observed in the number of Mini cells in the lymph nodes when Mini cells were transferred with an interval of 3 days relative to infection, we extended the gap between infection and adoptive transfer of transgenic cells to 7 days (Fig 5E). We quantified Mini cells in the LNs six days post antigen encounter/ adoptive cell transfer, and observed a 7-fold decrease in numbers of total Mini cells and about a 4-fold decreased number of Mini KLRG1^-^ cells in case of 7 days delayed transfer compared to day -1 transfer of Mini cells (Fig 5F). In addition to almost undetectable peak expansion, accumulation of Mini cells was markedly reduced throughout infection in the blood (Fig 5G), with a 5.7 to 6.3-fold reduction of total numbers of Mini cells in the lungs and spleen 130 days post infection (Fig 5H). Strikingly, this reduction in the number of Mini cells at day 130 of infection was within the range of the reduction of Mini cells established 6 days post antigen encounter. Taken together, these results suggest that the size of the early established pool of KLRG1^-^ MCMV-specific T cells is indicative for the size of memory inflation.

**Fig 6:**
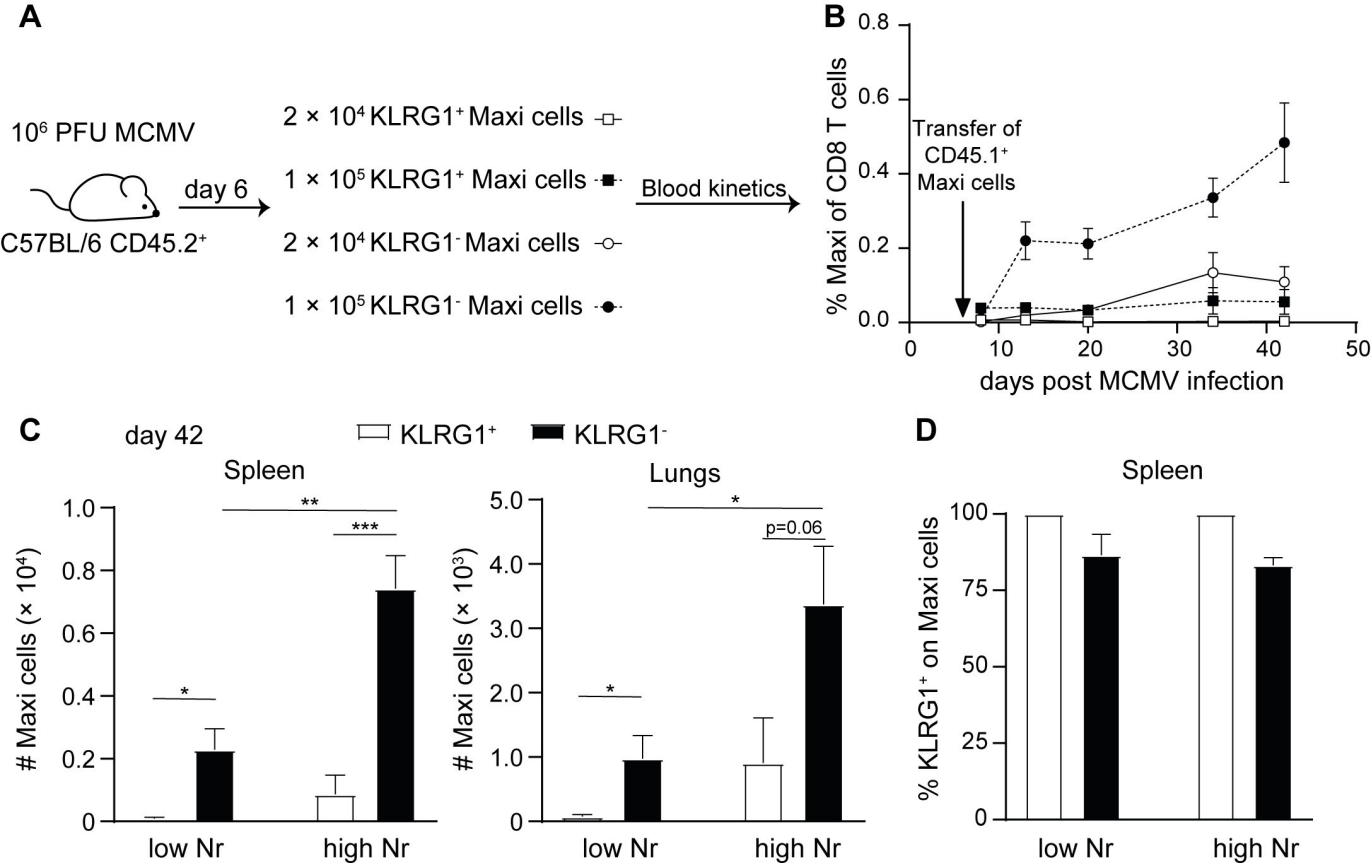
The number of early primed KLRG1^-^ MCMV-specific T cells correlates with the degree of memory inflation. (A) Experimental setup: CD45.1^+^ Maxi cells were transferred into naïve C57BL/6 mice one day prior to an i. v. infection with 10^6^ PFU MCMV-Δm157. On day 6 post infection, KLRG1^-^ and KLRG1^+^ Maxi cells were sorted and different numbers were transferred into infection matched recipients. (B) Kinetics of Maxi cells is shown in the blood, as percentage within the CD8 T cell population. (C) The total number of Maxi cells in the spleen and the lungs is shown at day 42 post infection corresponding to day 34 post Maxi cell transfer. (D) The percentage of Maxi cells expressing KLRG1 is shown for the spleen on day 42 post infection. All bar graphs represent mean + SEM of n = 5 mice representative of two independent experiments. Statistical analyses were performed using the unpaired two-tailed Student's *t* test (C), *p<0.05; **p<0.01; ***p<0.001.

To demonstrate that the number of KLRG1^-^ cells early in infection determines the size of the inflationary T cell pool, we adoptively transferred different numbers of KLRG1^-^ and KLRG1^+^ Maxi cells from day 6 infected mice into infection-matched recipients (Fig 6A), and longitudinally tracked the Maxi cells in the blood. Strikingly, only mice obtaining KLRG1^-^ cells accumulated Maxi T cells in the blood, whereas upon transfer of KLRG1^+^ cells, a small pool of Maxi cells was observed that did not increase in frequency in time (Fig 6B). Upon transfer of a low number of KLRG1^+^ cells, hardly any Maxi cells were detected in the circulation after resolution of acute MCMV infection. Moreover, when more KLRG1^-^ Maxi cells were transferred, also a higher number of Maxi cells was found in the spleen and the lungs at day 42 post infection (Fig 6C). The majority of Maxi cells that were derived from a KLRG1^-^ Maxi cell transfer expressed KLRG1 at this time point, indicating that KLRG1^-^ cells gave rise to the KLRG1^+^ cells (Fig 6D). These data support the notion that the number of KLRG1^-^ cells early in infection correlates to the degree of memory inflation.

### The magnitude of the inflationary T cell pool correlates to their protective capacity

**Fig 7:**
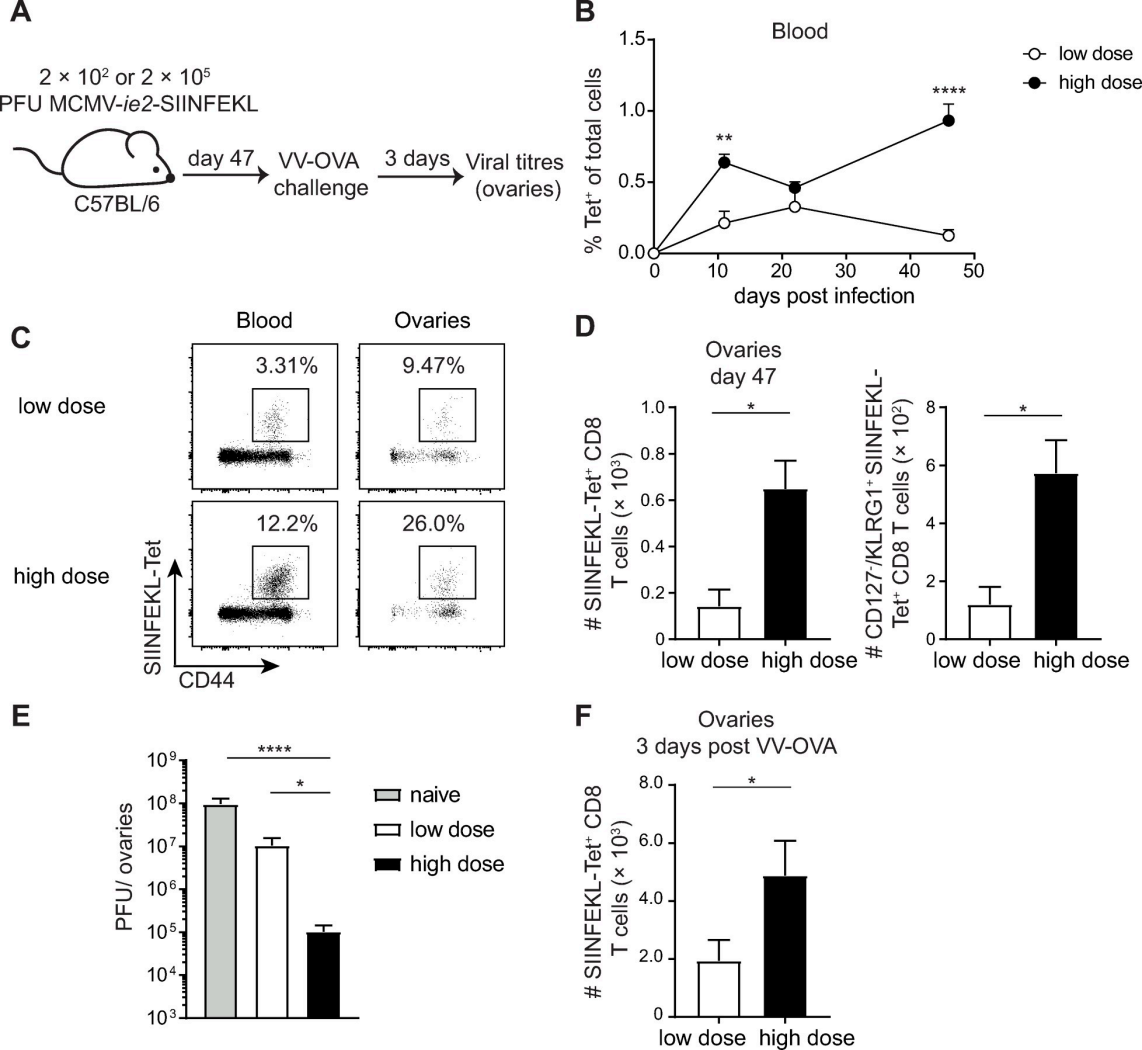
The number of inflationary T cells in peripheral tissues correlates with the protective capacity. (A) Experimental setup: Naïve C57BL/6 mice were infected with either 2 × 10^2^ (low dose) or 2 × 10^5^ (high dose) PFU MCMV-*ie2*-SIINFEKL. Mice were challenged i. p. at 47 days post MCMV infection with 2 × 10^7^ PFU VV-OVA. (B) Percentage of tetramer^+^ cells within total cells are shown in the blood as mean + SEM at indicated time points of n = 10 mice per group, pooled of two independent experiments. (C) Representative flow cytometry plots show the percentage of SIINFEKL-specific CD8 T cells determined by MHC Class I tetramer binding. Cells are gated on CD8 T cells and the percentage within the CD8 T cell pool is indicated. (D) Total numbers of SIINFEKL-specific CD8 T cells and CD127^-^/KLRG1^+^ expressing SIINFEKL-specific cells in the ovaries are shown at day 47 post MCMV-*ie2*-SIINFEKL infection as mean + SEM of n = 3 mice, shown is one representative experiment out of two independent experiments. (E) VV titres in the ovaries are shown 3 days post VV-OVA infection. (F) Total numbers of SIINFEKL-specific CD8 T cells in the ovaries at 3 days post-secondary challenge with VV-OVA. (E-F) Bar graphs represents mean + SEM of n = 5–12 mice, pooled from 2 independent experiments. Statistical analyses were performed using two-way ANOVA followed by Sidak's multiple comparisons test (B), the unpaired Student's *t* test (D, F) or the non-parametric Kruskal-Wallis test followed by Dunn's multiple comparisons test (E), *p<0.05; **p<0.01; ****p<0.0001.

Inflationary T cells can provide protection from an infection in peripheral tissues [25, 35]. Next, we addressed the question whether this protective capacity was also linked to the size of the inflationary T cell pool. For this purpose, mice were infected with two different doses of MCMV-*ie2*-SIINFEKL (Fig 7A), resulting in differences in the number of inflationary T cells that seed peripheral tissues [36]. Mice that were infected with a low dose of MCMV-*ie2*-SIINFEKL had reduced SIINFEKL-specific CD8 T cells in the blood and the ovaries, the organ of interest for VV challenge, as compared to mice that received a high dose infection (Fig 7B, Fig 7C, Fig 7D). The number of effector memory cells (CD127^-^/KLRG1^+^) in the ovaries was also diminished in low dose infected mice (Fig 7D). Similar to SIINFEKL-specific CD8 T cells in the blood, lungs and spleen, the majority of the SIINFEKL-specific cells in the ovaries had an effector-memory phenotype, documented by the expression of KLRG1 (S5A Fig), and did not express CD103 and CD69 (S5B Fig), markers associated with tissue residency. In addition, most of the cells were stained by injection of a fluorescently conjugated anti-CD8 antibody, implying that these cells were within or in close proximity to the vasculature (S5C Fig). These mice were subsequently challenged with VV-OVA and the viral burden was determined three days later. Mice that were infected with a low dose of MCMV-*ie2-*SIINFEKL only had a minor improved viral control compared to naïve mice (Fig 7E). However, mice that had received a high dose of MCMV-*ie*2-SIINFEKL had a 2 log-fold lower viral burden in the ovaries compared to low dose infected mice and a 3 log-fold lower viral load compared to naïve mice (Fig 7E). Consistent with the differences in the viral load, mice that had received a high dose of MCMV-*ie2*-SIINFEKL also had more SIINFEKL-specific CD8 T cells in the ovaries after secondary challenge with VV-OVA (Fig 7F). These results highlight the ability of inflationary CD8 T cells to control a peripheral virus infection, given that they are present in sufficient numbers in the circulation or the target organ prior to infection.

## Discussion

Memory inflation of CD8 T cells represents a process that is driven by low-level persisting antigen that is expressed sporadically and leads to reactivation of memory CD8 T cells, leading to high level accumulation of antigen-specific T_EM_-like CD8 T cells in the circulation and many peripheral organs [37, 38]. Murine and human CMV infection establish a condition that promotes such a process, by persistence of viral genomes in latently infected cells that are templates for transcription and translation of viral genes during sporadic viral reactivation events [39, 40]. In murine CMV infection, such reactivation events engage T_CM_ CD8 T cells with specificity for certain epitopes that are presented on non-hematopoietic cells and these CD8 T cells respond to this trigger by proliferation, effector memory differentiation and dissemination to peripheral organs where they establish a dynamic but stable pool of T_EM_ cells [13, 15]. The process of memory inflation is not only restricted to CMV infection, as it was also demonstrated during systemic HSV-1 infection, adenovirus infection [41] as well as human parvovirus B19 infection [42–44]. In case of MCMV infection, the fuelling of the inflationary T cell pool was shown to be independent of replication-competent CMV [45], but involved sensing of CMV antigens during latency by T_CM_ cells, leading to stable maintenance of the inflationary T cell pool in peripheral tissues [33]. However, the localisation of where the viral reactivation events and activation of T_CM_ takes place is still a matter of debate [15, 46] and cells harbouring (latent) MCMV genomes have been described in the lungs, kidney, liver, brain and salivary gland [6, 47–50]. In addition, a recent study identified CX3CR1^int^ CD8 T cells as possible source of proliferation-competent cells, which might contribute to the inflated T cell pool [51].

In this study, we sought to provide insights into the parameters that promote and limit inflation of CMV-specific CD8 T cells. We addressed the question whether there was a bias for TCR antigen avidity to be recruited into the inflationary pool of CMV-specific CD8 T cells. Our data show that inflationary T cells are almost exclusively consisting of high avidity CD8 T cells, although this selection was already apparent during initial clonal expansion. Most probably all low avidity CD8 T cells were outcompeted during acute and also latent MCMV infection. This is in contrast to human studies where in elderly individuals, low-avidity CD8 T cells are significantly contributing to the inflationary T cell pool during latency [28, 52]. Recently, low-avidity CMV-specific CD8 T cells were also described to be functional in human individuals with allogeneic stem cell transplantation [53]. These differences might be explained by the different time resolutions, and longer observation periods in mice might also reveal a contribution of low avidity clones [35].

It has been shown that regulatory T cells and IL-10 are restraining memory T cell inflation [54, 55]. Searching for other determinants that limit the 'size' of memory inflation, we ruled out a possible space (niche) limitation in peripheral tissues, for instance provided by IL-15 access in lung tissue [33]. By experimentally implementing a 50% reduction of the overall population of inflationary M38-specific CD8 T cells two to three weeks after priming had occurred, we did not observe a refilling of the vacated niche by the remaining endogenous M38-specific CD8 T cells. Instead, the overall M38-specific CD8 T cell pool remained at two-fold reduced frequencies and numbers for the entire period of more than 5 months of observation (Fig 2). As there might be competition for antigen at the level of the APC [21, 56], we ruled out that inflationary MCMV-specific CD8 T cells with other specificities occupied the newly available space. Thus, we concluded that there must be another critical determinant for the size of memory inflation which is likely to act early during MCMV infection. Indeed, we identified the number of primed cells, and in particular the number of KLRG1^-^ cells, to be indicative for the size of the inflationary T cell pool during MCMV latency. These results are in line with a previous report that showed that the inflationary T cell pool is maintained at least in part by T cells primed early in infection [13]. Transferring distinct numbers of T_CM_ cells with inflationary specificity recapitulated these results. In models of acute infection it has been shown that KLRG1^-^ cells have a higher probability to feed into the memory pool [34], making it likely that these cells also give rise to the T_CM_ cells in MCMV infection. Thus, the number of central memory T cells that is able to sense viral reactivation events is one feature that drives memory inflation, but more factors promote T cell inflation as well [57]. This for instance includes the amount of latent viral genomes present in non-hematopoietic cells that is also related to the viral inoculum dose and the amount of latent viral genomes in the spleen determined by the route of infection and by immune evasion strategies [15, 36, 58, 59]. Also CD4 T cell help, co-stimulatory signals mediated via 4-1BB, CD27 and OX40, and IL-2 mediated signals have been shown to promote T cell inflation [38, 60–66].

The interest of using CMV-based vectors for vaccination purposes is rising and CMV-vectors encoding antigens of heterologous viruses have already shown promising results in experimental studies [26, 67–71]. The success of these CMV based vaccines is due to the induction of the large pool of effector memory T cells in the circulation and peripheral tissues which requires optimal epitope expression in latently infected, non-hematopoietic cells and its availability for processing by the constitutive proteasome [15, 72]. Also here we show a prominent role of inflationary CD8 T cells in mediating immediate protection from a viral challenge that needs to be rapidly controlled in peripheral tissues [25, 35], and we show that this protective capacity is directly correlated to the size of the inflationary T cell pool. Similar results were shown recently where CMV-based vectors were used in a prophylactic manner to control tumour growth [73]. Although central memory T cells have a superior proliferation capacity as compared to effector memory T cells [74, 75], the activation of these T_CM_ cells is rather a slow process as this requires antigen to be transported to secondary lymphoid tissues, reactivation of T cells and local expansion. Furthermore, the expanded cells have to migrate back to the initial site of infection. As inflationary T cells are either within or in close contact with the vasculature [33, 46, 76, 77], they have the ability to continuously seed non-lymphoid tissues in high numbers and therefore they are already positioned at the site where viral replication needs to be controlled. Related to this it would be interesting to compare the protective capacity of inflationary T cells with memory T cells that are confined to peripheral tissue, such as tissue resident memory cells [37]. Core 2 O-glycan synthesis, which is required to generate functional ligands for E- and P-selectins and therefore promotes trafficking into inflamed tissue in an antigen independent manner, is highly active in T_CM_ cells but limited in T_EM_ cells [78]. Recently it was shown that when MCMV infected mice received a subsequent Vaccinia challenge in the skin, non-inflationary T cells infiltrated the skin much better as compared to inflationary T cells [78]. Although this challenge was performed in an antigen independent manner, on a per-cell basis, T_CM_ cells might be better in entering non-lymphoid tissues as compared to T_EM_ cells. However, inflationary T cells are already at the location in large numbers and it is likely that they will control a peripheral infection quicker due to their numerical advantage. Moreover, we show that if we experimentally diminish the amount of inflationary T cells in a peripheral organ, then also their protective capacity is diminished. Thus, the immediate protective capacity of the inflationary T cell pool is directly correlated to the number of T cells in circulation and / or specific tissues. As the limit for memory inflation is already determined early during infection, changing the balance between early primed KLRG1^-^ and KLRG1^+^ cells is of interest for the efficacy of CMV-based vaccine vectors as well. Combined, these data emphasise the importance of inflationary T cells in the context of CMV-based vaccine vectors and their ability to protect from a virus challenge in peripheral tissues.

## Material and methods

### Ethics statement

This study was conducted in accordance to the guidelines of the animal experimentation law (SR 455.163; TVV) of the Swiss Federal Government. The protocol was approved by Cantonal Veterinary Office of the canton Zurich, Switzerland (Permit number 127/2011, 146/2014, 114/2017).

### Mice

Wild-type C57BL/6J were purchased from Janvier Elevage (Le Genest Saint Isle, France). C57BL/6N-*Tg(TcraM38*,*TcrbM38)329Biat* (Maxi) [15], C57BL/6N-*PtprcaTG(TcrbM38)330Biat* (Mini) [15], C57BL/6-Tg(TcraTcrb)1100Mjb/J (OT-I) [79] and B6.(PL-Thy1a;B6;129P2/OlaHsd)-Tg((TcrOVA)1Zhn/UniL) (OT-III) [29] mice were housed and bred in specific pathogen-free facilities at the Eidgenössische Technische Hochschule (ETH) Hönggerberg. Maxi transgenic (Ly5.1^+^) express a TCR (Vβ10Jβ2.1/Vα4Jα13) specific for the MCMV peptide M38_316-323_ [15]. Mini transgenic (Ly5.1^+^) mice express the TCR Vβ10Jβ2.1 chain of the Maxi TCR, harbouring M38_316-323_-specific CD8 T cells at roughly 10%. OT-I transgenic (Ly5.1^+^) mice express a TCR specific for the ovalbumin peptide OVA_257-264_ (SIINFEKL) [79]. OT-III transgenic (Thy1.1^+^) mice express a low avidity TCR specific for the SIINFEKL epitope [29]. Female or male mice were used at 6–12 weeks of age and sex-matched within all experiments.

### Viruses and infections

Recombinant MCMV lacking m157 (MCMVΔm157) was previously described and is referred to as MCMV in this study [65]. Recombinant MCMV expressing OVA_257-264_ SIINFEKL peptide within the *ie2* gene was produced as described [21], contains the m157 gene, and is referred to as MCMV-*ie2*-SIINFEKL. MCMV strains were propagated on MEFs [80] or M2-10B4 cells [81] as previously described. Virus titres in organs were determined by standard plaque-forming assays on M2-10B4 cells as previously described [81]. Infections were performed intravenously with 1–5 × 10^6^ PFU MCMV, or 2 × 10^5^ (high dose) or 2 × 10^2^ (low dose) PFU MCMV-*ie2*-SIINFEKL. Recombinant Vaccinia virus (Western Reserve) expressing Ovalbumin protein (VV-OVA) inserted into the thymidine kinase gene was grown on BSC40 cells and was provided by Dr. P. Klenerman. MCMV-*ie2*-SIINFEKL infected female hosts were challenged i. p. with 2 × 10^7^ PFU VV-OVA. VV titres were analysed in the ovaries three days post challenge by standard plaque-forming assay on BSC40 cells as previously described [82].

### Adoptive transfer

CD8 T cells from naïve Maxi, Mini, OT-I or OT-III mice were purified from splenocytes using anti-CD8α MACS beads (Miltenyi Biotech) according to the manufacturer's instructions. 10^4^ purified Maxi CD8 T cells or 2 × 10^5^ Mini, OT-I or OT-III CD8 T cells were adoptively transferred into recipient mice one day prior to infection, unless otherwise stated. High and low avidity Mini cells were sorted from the spleen of naïve Mini mice according to tetramer staining, and 10^5^ sorted high or low avidity Mini CD8 T cells were transferred into recipient mice. Maxi T_CM_ cells were isolated from the spleen and LNs of infected C57BL/6 mice after at least 60 days of MCMV infection, and sorted according to the expression of CD127 and CD62L. Sorted cells were labelled with cell proliferation dye-eFluor450 (Life technologies) according to manufacturer's protocol. Maxi KLRG1^-^ and KLRG1^+^ cells were isolated from the spleen and LNs of infected C57BL/6 mice at day 6 post MCMV infection. Maxi cells isolated from infected mice were enriched before sorting by depletion of CD4 T cells and B cells using biotinylated CD4 (GK1.5) and B220 (RA3-6B2) antibodies and MojoSort Streptavidin nanobeads (BioLegend). For all sorting experiments, a BD FACSAria Sorter was used.

### Lymphocyte isolation, peptide restimulation and surface staining

Lymphocytes were isolated from spleen and lungs as described before [83]. Cells were isolated from ovaries by mincing the tissue through a 70 μm cell strainer. Before lungs and ovaries were removed, mice were perfused with PBS. Blood samples were obtained from the tail vein. Red blood cells were lysed using ACK lysis buffer for 1 minute at room temperature. Surface staining of cells was performed for 20 min at room temperature in PBS supplemented with 2% FCS. For i. v. labelling of CD8 T cells, 5 μg of a fluorescently conjugated anti-CD8α antibody was injected i. v. 3 minutes prior to euthanasia. For peptide restimulations, cells were incubated with 1 μg/ml peptide in the presence of 20 μM Monensin A (Sigma Aldrich) for 6 hours at 37° C. Cell surface staining was performed as described above and cells were fixed with 1% PFA for 20 minutes. Cells were permeabilized using 2x BD lysis buffer (BD Biosciences) containing 0.05% Tween 20 (Sigma Aldrich) for 10 minutes. Intracellular staining was performed at room temperature for 20 minutes. Multiparametric flow cytometric analysis was performed using LSRII flow cytometer (BD Biosciences) and FACSDiva software. Data was analysed using FlowJo software (Tree Star).

### Antibodies and tetramers

APC- or PE-conjugated MHC class I tetramers were generated as described before [84]. Fluorophore-conjugated antibodies were purchased from BioLegend (Lucerna Chem) or eBiosciences (Thermo Fisher Scientific). The following antibodies were used for Flow cytometry: anti-CD8α (53–6.7), anti-CD8β (53–5.8), anti-CD45.1 (A20), anti-CD45.2 (104), anti-CD90.1 (Ox-7), anti-CD90.2 (30-H12), anti-CD62L (MEL-14), anti-CD44 (IM7), anti-KLRG-1 (2F1), anti-CD127 (A7R34), anti-CD69 (H1.2F3), anti-CD103 (2E7), anti-CD25 (3C7) and anti-CD4 (RM4-5). Live/Dead Fixable near-IR (Life Technologies) dead cell stain was used to exclude dead cells. Transgenic Mini and Maxi cells were identified by gating on CD8α^+^ CD45.1^+^ M38-Tet^+^ cells. OT-I cells were identified by gating on CD8α^+^ CD45.1^+^ cells and OT-III were identified by gating on CD8α^+^ CD90.1^+^ cells.

### Statistical analysis

Statistical significance was determined using GraphPad Prism (La Jolla) and statistical tests are indicated in each figure.

## Supporting information

S1 Fig Low avidity OT-III CD8 T cells do not contribute to the inflationary T cell pool. Experimental setup: 2 × 10^5^ OT-I or OT-III CD8 T cells were transferred into naïve C57BL/6 mice one day prior to i. v. infection with 2 × 10^5^ PFU MCMV-*ie2*-SIINFEKL. Percentages of transgenic CD8 T cells (A) and endogenous SIINFEKL-Tet^+^ CD8 T cells (B) were measured in the blood. (C) Percentages of transgenic CD8 T cells in the lungs and spleen at day 60 post infection are shown. (D) Percentages of KLRG1^+^ CD127^-^ cells of transgenic CD8 T cells in the lungs and spleen at day 60 post infection are shown. (E) MFI of H2-K^b^ M38 tetramer of OT-I and OT-III cells in the spleen and lungs at day 60 post infection is shown. (A-E) All data is shown as mean + SEM, representative of 2 independent experiments with 5 mice per group, *p<0.05; **p<0.01; ***p<0.001, ****p<0.0001. Statistical analyses were performed using two-way ANOVA followed by Sidak's multiple comparisons test (A-B) or the non-parametric Mann-Whitney *U* test (C-E).
(TIF)

S2 Fig Space is not limiting the size of the inflationary T cell pool. Experimental setup: Naïve female C57BL/6 mice were transferred with 2 × 10^5^ male or female Mini CD8 T cells one day prior to an i. v. infection with 5 × 10^6^ PFU MCMVΔm157. (B) Percentages of M38-specific CD8 T cells in mice transferred with male or female Mini cells were measured in the blood and (C) percentages of M38-specific CD8 T cells in the lungs and spleen at day 130 post infection are shown. (D) Percentages of IFN-γ^+^ of CD8 T cells upon peptide restimulation are shown in the lungs at day 130 post infection. (B-D) All data is shown as mean + SEM, representative of 2 independent experiments with 5 mice per group, *p<0.05; **p<0.01. Statistical analyses were performed using two-way ANOVA followed by Sidak's multiple comparisons test (B, D) or the unpaired two-tailed Student's *t* test (C).
(TIF)

S3 Fig Increasing the precursor frequency results in correspondingly increased population size of the inflated CD8 T cell pool. (A) Experimental setup: 10^3^, 10^4^ or 10^5^ CD45.1^+^ OT-I CD8 T cells were transferred into naïve C57BL/6 mice one day prior to infection with 2 × 10^5^ PFU MCMV-*ie2*-SIINFEKL infection. (B) Percentages of OT-I cells within CD8 T cells were determined in the blood at indicated time points post infection. **p<0.01, ****p<0.0001. Statistical analyses were performed using the two-way ANOVA followed by Sidak's multiple comparisons test, significance is only indicated for the final time point.
(TIF)

S4 Fig Late recruited Mini cells adopt a KLRG1^-^ phenotype. Experimental setup: 2 × 10^5^ CD45.1^+^ Mini CD8 T cells were adoptively transferred into naïve C57BL/6 mice one day prior, or three days after an i. v. infection with 5 × 10^6^ PFU MCMVΔm157. The phenotype of Mini cells in the lymph node was determined 6 days post antigen encounter. Cells are gated on CD45.1^+^.
(TIF)

S5 Fig SIINFEKL-specific CD8 T cells in the ovaries have predominantly an effector memory phenotype. Naïve C57BL/6 mice were infected with 2 × 10^5^ PFU MCMV-*ie2*-SIINFEKL. At day 30 post infection, the phenotype of SIINFEKL-specific CD8 T cells was determined in different organs. Representative flow cytometry plot shows cell surface expression of CD127 and KLRG1 (top row), CD69 and CD103 (middle row), and i.v. labelling for CD8 (bottom row), on SIINFEKL-specific CD8 T cells.
(TIF)
