# Supplementary figures and images for: Early primed KLRG1- CMV-specific T cells determine the size of the inflationary T cell pool
Source: PLoS Pathog. 2019 May 13;15(5):e1007785. doi: 10.1371/journal.ppat.1007785 (PMC6532941; doi:10.1371/journal.ppat.1007785)

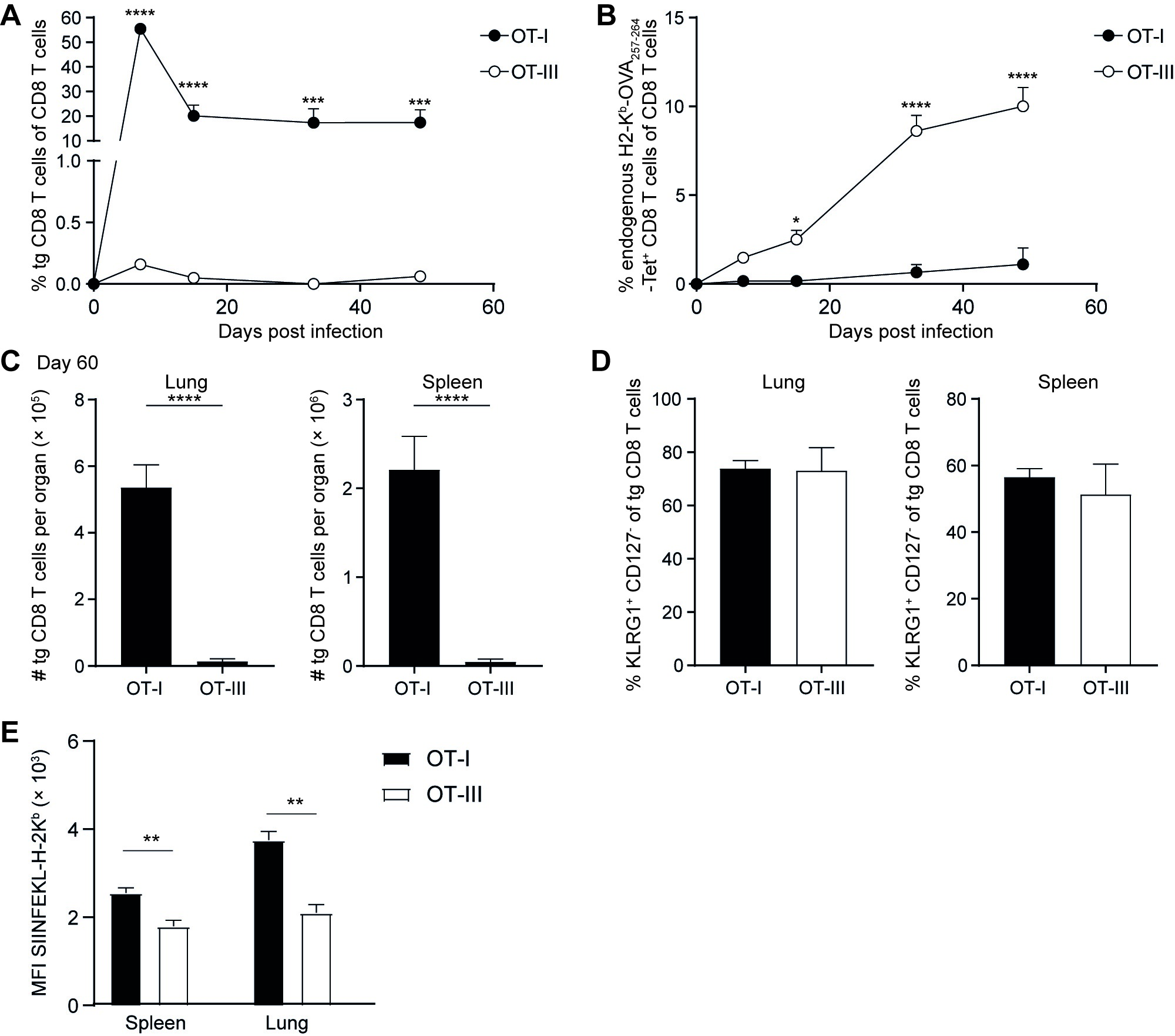

Supplement: S1 Fig — Experimental setup: 2 × 10^5^ OT-I or OT-III CD8 T cells were transferred into naïve C57BL/6 mice one day prior to i. v. infection with 2 × 10^5^ PFU MCMV-ie2-SIINFEKL. Percentages of transgenic CD8 T cells (A) and endogenous SIINFEKL-Tet+ CD8 T cells (B) were measured in the blood. (C) Percentages of transgenic CD8 T cells in the lungs and spleen at day 60 post infection are shown. (D) Percentages of KLRG1+ CD127- cells of transgenic CD8 T cells in the lungs and spleen at day 60 post infection are shown. (E) MFI of H2-Kb M38 tetramer of OT-I and OT-III cells in the spleen and lungs at day 60 post infection is shown. (A-E) All data is shown as mean + SEM, representative of 2 independent experiments with 5 mice per group, *p<0.05; **p<0.01; ***p<0.001, ****p<0.0001. Statistical analyses were performed using two-way ANOVA followed by Sidak's multiple comparisons test (A-B) or the non-parametric Mann-Whitney U test (C-E). (TIF) [file ppat.1007785.s001.tif]

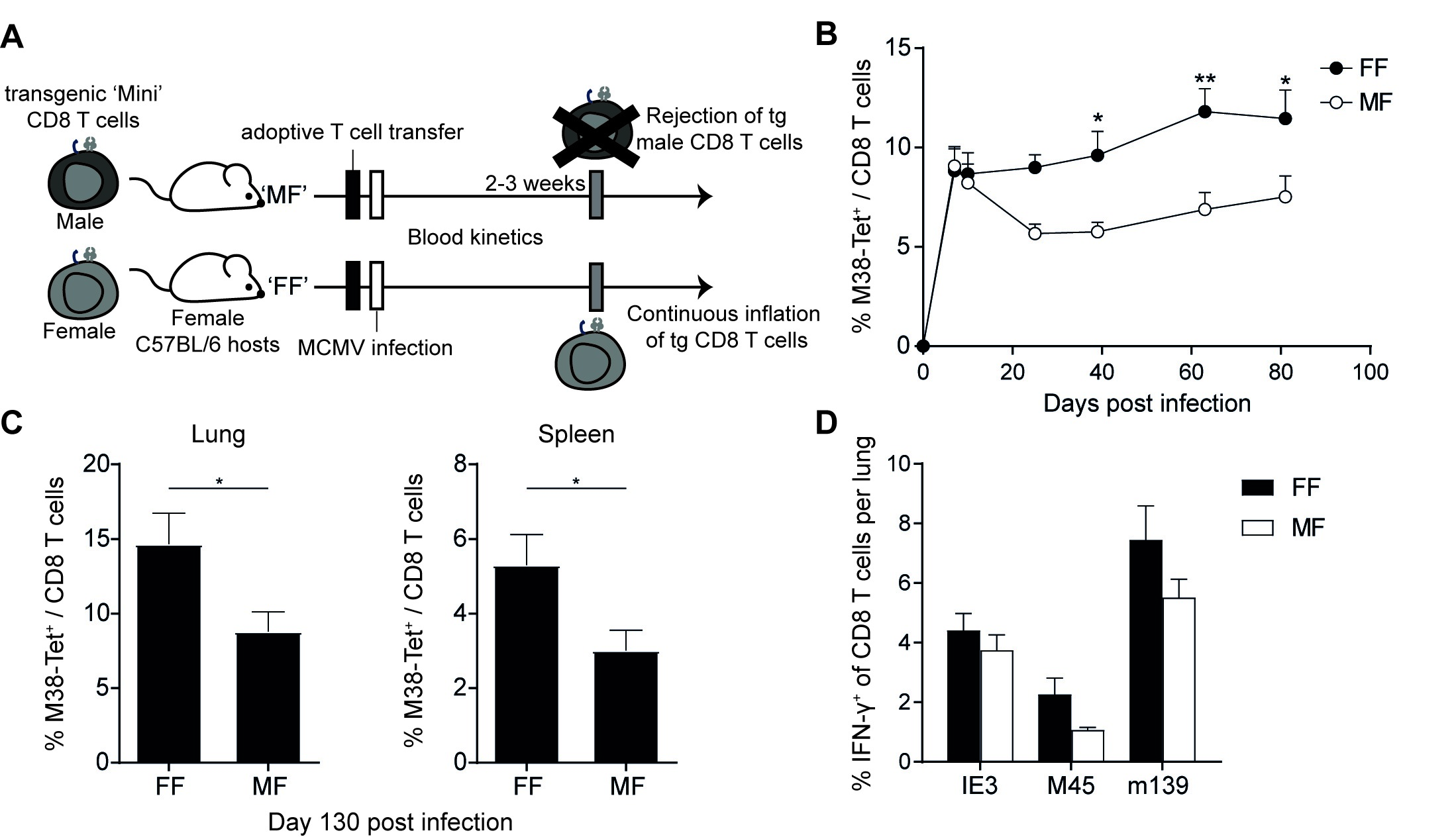

Supplement: S2 Fig — Experimental setup: Naïve female C57BL/6 mice were transferred with 2 × 10^5^ male or female Mini CD8 T cells one day prior to an i. v. infection with 5 × 10^6^ PFU MCMVΔm157. (B) Percentages of M38-specific CD8 T cells in mice transferred with male or female Mini cells were measured in the blood and (C) percentages of M38-specific CD8 T cells in the lungs and spleen at day 130 post infection are shown. (D) Percentages of IFN-γ+ of CD8 T cells upon peptide restimulation are shown in the lungs at day 130 post infection. (B-D) All data is shown as mean + SEM, representative of 2 independent experiments with 5 mice per group, *p<0.05; **p<0.01. Statistical analyses were performed using two-way ANOVA followed by Sidak's multiple comparisons test (B, D) or the unpaired two-tailed Student's t test (C). (TIF) [file ppat.1007785.s002.tif]

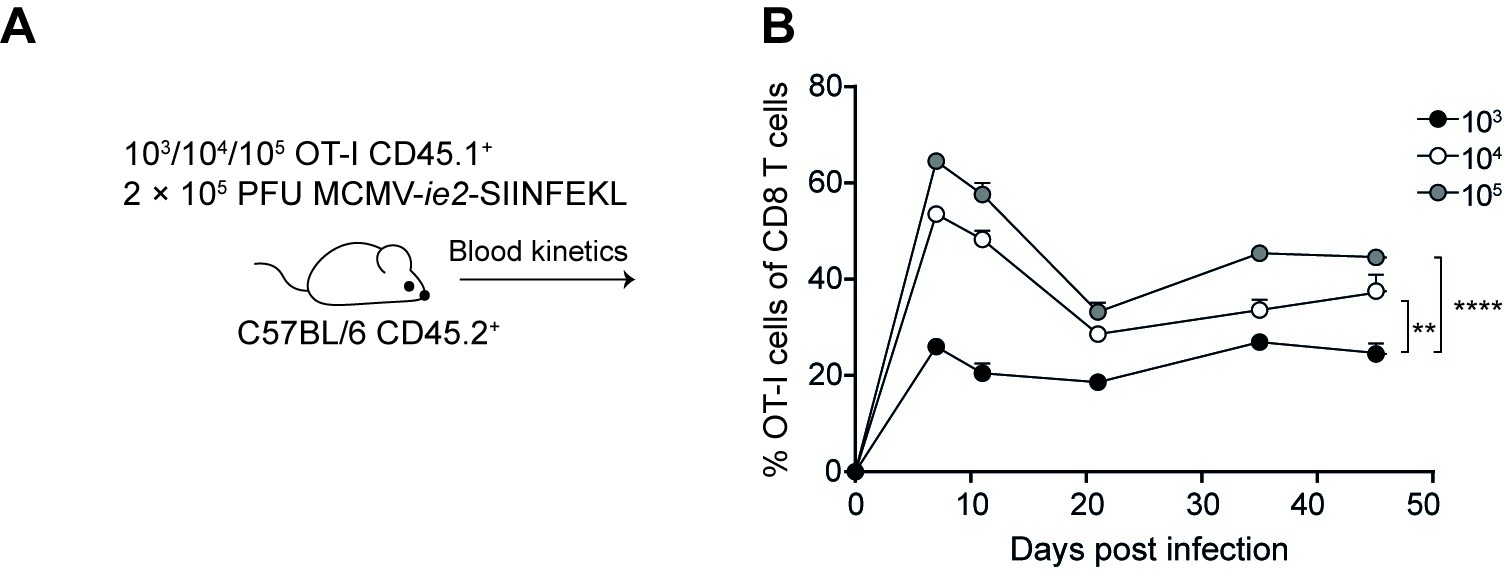

Supplement: S3 Fig — (A) Experimental setup: 103, 104 or 105 CD45.1+ OT-I CD8 T cells were transferred into naïve C57BL/6 mice one day prior to infection with 2 × 10^5^ PFU MCMV-ie2-SIINFEKL infection. (B) Percentages of OT-I cells within CD8 T cells were determined in the blood at indicated time points post infection. **p<0.01, ****p<0.0001. Statistical analyses were performed using the two-way ANOVA followed by Sidak's multiple comparisons test, significance is only indicated for the final time point. (TIF) [file ppat.1007785.s003.tif]

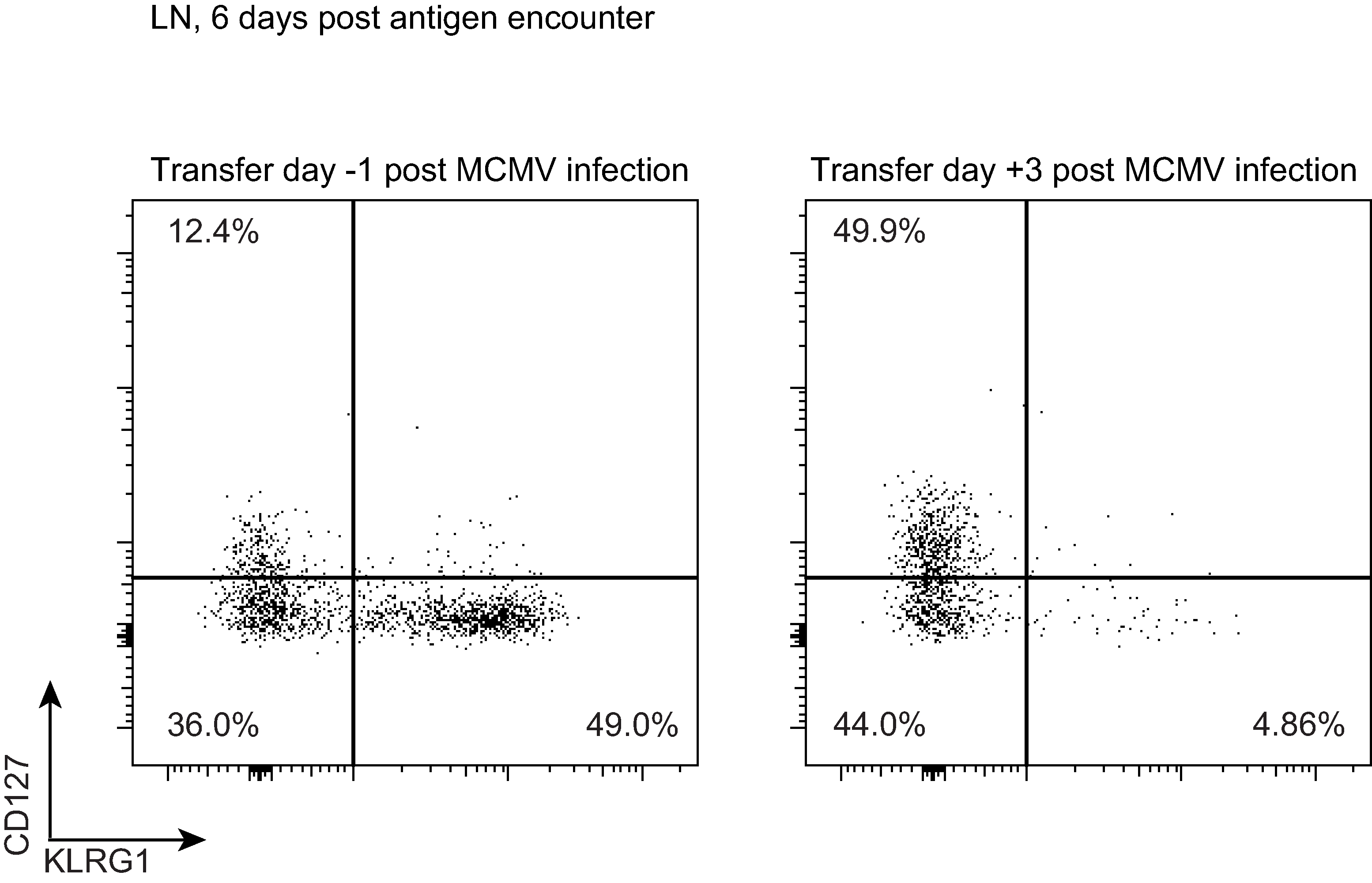

Supplement: S4 Fig — Experimental setup: 2 × 10^5^ CD45.1+ Mini CD8 T cells were adoptively transferred into naïve C57BL/6 mice one day prior, or three days after an i. v. infection with 5 × 10^6^ PFU MCMVΔm157. The phenotype of Mini cells in the lymph node was determined 6 days post antigen encounter. Cells are gated on CD45.1+. (TIF) [file ppat.1007785.s004.tif]

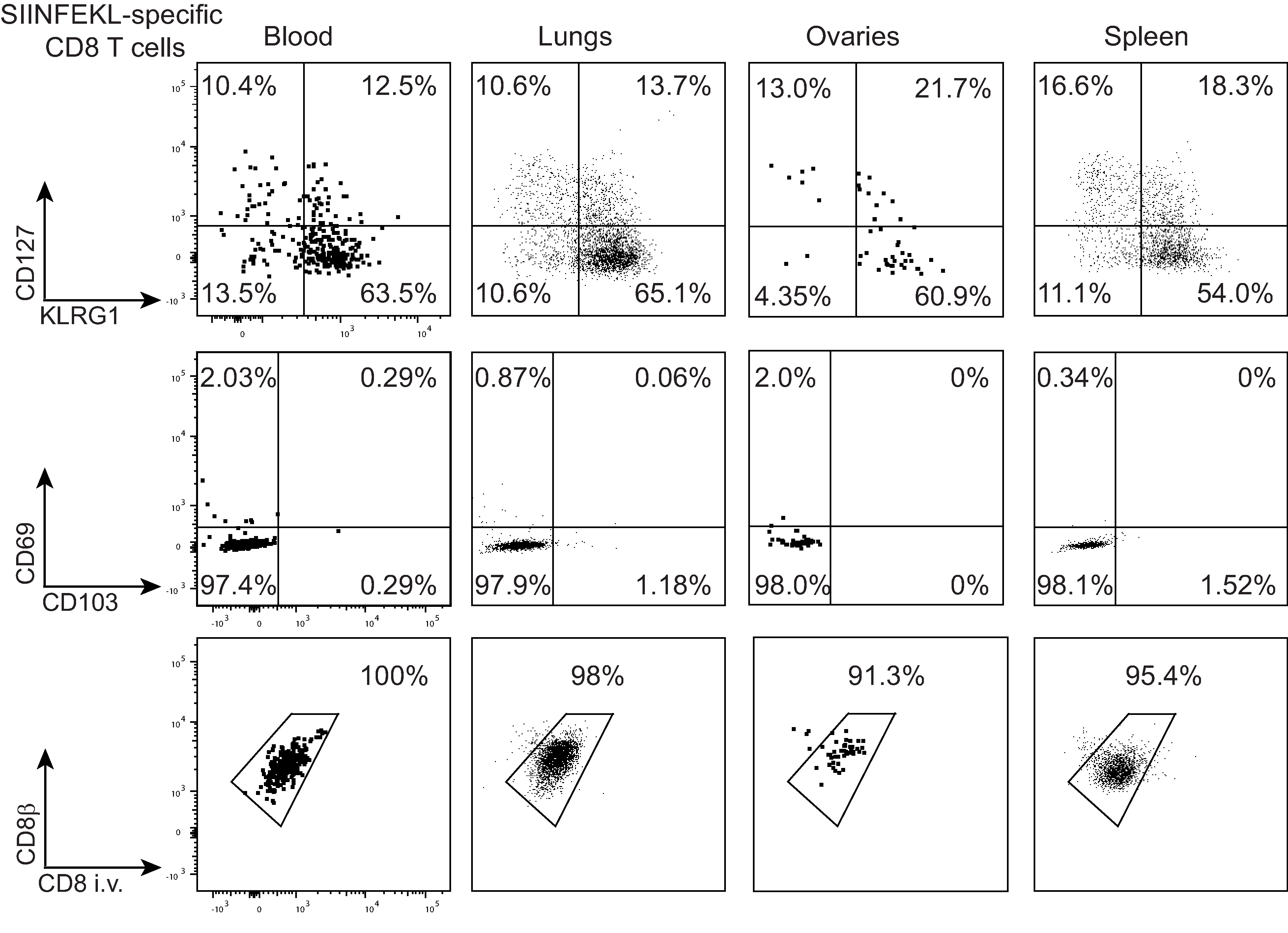

Supplement: S5 Fig — Naïve C57BL/6 mice were infected with 2 × 10^5^ PFU MCMV-ie2-SIINFEKL. At day 30 post infection, the phenotype of SIINFEKL-specific CD8 T cells was determined in different organs. Representative flow cytometry plot shows cell surface expression of CD127 and KLRG1 (top row), CD69 and CD103 (middle row), and i.v. labelling for CD8 (bottom row), on SIINFEKL-specific CD8 T cells. (TIF) [file ppat.1007785.s005.tif]
